# Defective antigen presentation by monocytes in ESRD patients not responding to hepatitis B vaccination: impaired HBsAg internalization and expression of ICAM-1 and HLA-DR/Ia molecules

**DOI:** 10.1155/S0962935195000093

**Published:** 1995-01

**Authors:** J. Stachowski, C. Barth, M. Pollok, J. Michałkiewicz, K. Madaliński, J. Maciejewski, C. A. Baldamis

**Affiliations:** 1 Department of Nephrology 5th Clinic of Internal Medicine University of Cologne Germany; 2 Department of Nephrology 2nd Clinic of Children's Diseases University School of Medical Sciences Poznan Poland; 3 Department of Immunology Children's Memorial Hospital Warsaw Poland

## Abstract

This study was undertaken to evaluate the monocyte function of uraemic non-responders to hepatitis B vaccination. Therefore, some parameters concerning antigen processing by monocytes (Mo) as antigen presenting cells (APC) were analysed. It was found that in uraemic non-responders, (1) the internalization of HBsAg by monocytes was significantly decreasjed—HBsAg complexed with specific IgG or as immune complex isolated from patients is better internalized compared with free HBsAg; (2) during antigen presentation the expression of adhesion (ICAM-1) and accessory (HLA-DR/Ia) molecules was significantly decreased in uraemic patients, especially in non-responders; and (3) impaired internalization of HBsAg as well as a decrease in ICAM-1 and HLA-DR/Ia expression, correlated well with the blunted proliferation of CD4^+^ T cells stimulated by autologous monocytes induced by HBsAg.


Mediators of Inflammation 4, 49–54 (1995)

### Defective antigen presentation by monocytes in ESRD patients not responding to hepatitis B vaccination: impaired HBsAg internalization and expression of ICAM-1 and HLA-DR/Ia molecules

J. Stachowski,[1,2,CA] C. Barth,[1] M. Pollok,[1] J. Michałkiewicz,[3] K. Madaliński,[3] J. Maciejewski[2] and C. A. Baldamis[1]

[1]Department of Nephrology, 5th Clinic of Internal Medicine, University of Cologne, Germany; [2]Department of Nephrology, 2nd Clinic of Children's Diseases, University School of Medical Sciences, Poznan, Poland; [3]Department of Immunology, Children's Memorial Hospital, Warsaw, Poland

[CA] Corresponding Author

**This study was undertaken to evaluate the monocyte function of uraemic non-responders to hepatitis B vaccination. Therefore, some parameters concerning antigen processing by monocytes (Mo) as antigen presenting cells (APC) were analysed. It was found that in uraemic non-responders, (1) the internalization of HBsAg by monocytes was significantly decreased—HBsAg complexed with specific IgG or as immune complex isolated from patients is better internalized compared with free HBsAg; (2) during antigen presentation the expression of adhesion (ICAM-1) and accessory (HLA-DR/Ia) molecules was significantly decreased in uraemic patients, especially in non-responders; and (3) impaired internalization of HBsAg as well as a decrease in ICAM-1 and HLA-DR/Ia expression, correlated well with the blunted proliferation of CD4$^+$ T cells stimulated by autologous monocytes induced by HBsAg.**

**Key words**: Adhesion molecules, Antigen presentation, HBV vaccination, Monocytes, Uraemia

### Introduction

After immunization with hepatitis B (HBV) vaccine, 95% of healthy individuals develop antibodies against hepatitis B surface antigen (HBsAg). In contrast, only 60–70% of dialysis patients show a seroconversion even after additional injections of the vaccine.[1] Various attempts have been made to enhance antibody production and to clarify the causes of this lack of response. Recent studies have shown that in uraemic non-responders to HBV vaccination, T lymphocytes do not receive the monocyte-derived signals that are essential for adequate antigen presentation.[1] *In vitro*, this defect can partially be counteracted by addition of monocytes from healthy donors[2–4] or simultaneous supplementation of IL-2, IL-1, IL-6 and IFN-γ.[1,5] In the present study we tested the monocyte internalization (phagocytic) capacity for HBsAg, as well as adhesion (ICAM-1/intercellular adhesion molecule-1) and accessory molecule (HLA-DR/Ia) expression on monocytes induced by HBsAg. The parameters examined were correlated with responsiveness to HBV vaccination in patients undergoing intermittent haemodialysis (ESRD patients).

### Materials and Methods

*Patients:* This study was carried out according to the Declaration of Helsinki, and the informed consent of each patient was obtained. Thirty haemodialysis patients (20 males, ten females) who had not produced antibodies after at least four injections of H-B-Vax-D[6] vaccine (U-NR, uraemic non-responders) and ten uraemic haemodialysis patients who had responded to the vaccine (U-R, uraemic responders; anti-HBs antibody titre higher than 10 U/l) were included in the study. Ten healthy volunteers who had responded well to HBV vaccination (C-R, control responder; anti-HBs antibody higher than 10 U/l) and ten healthy blood donors (C-NR, control non-responders; Institute of Transfusiology, University of Cologne) not responding to vaccination were selected as control groups for the trial. The mean age of patients was 25.6 years (range 8–28 years), the mean duration of haemodialysis was 3.1 years (range 1–6 years). The mean age of controls was 28.5 years (range 21–35 years). None of these subjects had the hepatitis B virus infection before introduction to the vaccination schedule (HBsAg-negative, anti-HBc/IgG and anti-HBc/IgM-negative).

© 1995 Rapid Communications of Oxford Ltd

*Vaccination:* Subjects were vaccinated (Gen-H-B-Vax-D; Merck Sharp & Dohme) in accordance with the standard schedule for hepatitis B vaccination (four doses of 40 μg given intramuscularly, deltoid or quadriceps muscle) at 0, 1, 2 and 6 months. Non-responsiveness was defined if the antibody titre after vaccination reached less than 10 U/l 6–8 weeks following the last booster injection.

*Purification of monocytes and T cells:* Heparinized blood was drawn before the haemodialysis session, after the longest interdialytic interval in order to minimize a direct influence of the previous haemodialysis procedure. Peripheral blood mononuclear cells (PBMC) were obtained by density gradient centrifugation using Ficoll–Paque (Lymphoflot, Biotest Diagnostics).

Monocytes were prepared from PBMC by adherence to glass Petri dishes. Cells at 2 × $10^6$/ml were incubated in a 1 ml volume for 2 h at 37°C in flat-bottom 24-well culture plates (Nunclon, InterMed Nunc, Denmark). Adherent cells consisting of about 95% pure monocytes as assessed by measurement of CD14 molecule expression (flow cytometry), were used as APC.[4]

Non-adherent cells were removed by washing. $CD4^+$ T cells were prepared from non-adherent cells by using the E-rosetting technique with subsequent passage over the nylon wool column (to deplete residual B cells and monocytes). Then the $CD4^+$ T cells were purified by negative selection by using a panning technique to deplete contaminating Ia positive cells and CD8 cells. Flow cytometry analysis of the resultant $CD4^+$ population showed that contamination with residual B cells (CD19), monocytes (CD14) and $CD8^+$ cells was less than 1%.

In order to exclude an influence of nonspecific monocyte activation by adherence, all experiments were performed simultaneously with monocytes originating from uraemic patients and controls (non-responders, responders).

*Internalization activity of monocytes:* Internalization analysis of HBsAg by monocytes was based on the technique used in the Phagotest (Orpegen, Med-Molekularbiologische Forschungsanstalt GmbH, D-6900 Heidelberg), where either free HBsAg or immune complexes containing HBsAg were used instead of opsonized FITC-conjugated *E. coli*. Labelling of protein molecules with FITC is based on the phenomenon that some amino acids, mainly lysine, can bind fluorochrome molecules (FITC).[7–11] The following HBsAg preparation were used in our experiments: (1) free HBsAg (ad or ay subtypes, Biodesign-International); (2) HBsAg conjugated *in vitro* with specific IgG (anti-HBs, goat IgG, B124046, Biodesign-International); affinity of anti-HBs to HBsAg ranged between 2 × $10^6$ and 9 × $10^7$ mol/l;[12] and (3) specific immune complexes containing HBsAg (Ic–HBsAg–IgG) isolated from sera of children suffering from glomerulonephritis associated with HBV infection.[13] Sera were fractionated by means of a two-stage chromatography system with Sephadex G-200 filtration followed by Sepharose 6B, using calibrated columns.[14]

A solution of fluorescein 5-isothiocyanate (FITC, 3 mg, isomer I F7250, Sigma, $C_{21}H_{11}NO_2S$) in borate buffer (pH 9.25, 100 ml) was used for labelling HBsAg-containing proteins.[7,8] The ratio of the fluorochrome molecule (F) conjugated to the protein molecule (P, HBsAg preparation) (F/P ratio) was estimated photometrically in the FITC–HBsAg solution. The absorbance at 495 nm and at 280 nm was determined using double-distilled water as a blank. The results were calculated according to Equation 1.

$$\frac{F}{P} = \frac{6.85 \times E_{495}}{E_{280} - 0.35 \times E_{495}} \quad (1)$$

The internalization of the HBsAg-preparation conjugated with FITC was assayed using a flow cytometer (FACScan; Becton–Dickinson) using the live gate, similar to that in the Phagotest procedure. FITC positive cells gated as monocyte clusters were proportional to the monocyte number that had phagocytosed HBsAg. Fluorescence intensity, however, was proportional to the number of internalized HBsAg molecules.

*Antigen presenting function of monocytes:* The antigen presenting function of monocytes (Mo) was tested by preparing HBsAg-pulsed monocytes (P-Mo, 2 × $10^4$ cells/ml), which were subsequently cultured with autologous $CD4^+$ T cells (1 × $10^5$/ml) for 6 days. P-Mo were prepared by placing 1 × $10^6$ cells/ml in polysulphon tubes, irradiating (2 500 rad) the cells and then incubating them with or without HBsAg, at a concentration of 100 ng/ml, in complete medium for 18 h. Irradiated monocytes incubated with HBsAg internalize and digest this antigen which is re-expressed on the surface of the cell in association with HLA class II (MHC) and is presented onto T cells during 6-day culture.[3,15,16] These irradiated monocytes are not able to transform by cell division during the culture, but they are able to perform the function of surface receptor expression and monokine synthesis; T lymphocytes then respond to the altered surface of monocytes by proliferating. Only free HBsAg (Biodesign-International) was used for the *in vitro* stimulation in order to avoid the nonspecific monocyte activation of IgG contained in immune complexes via its Fc receptor.

*Proliferation assays:* The proliferative response of $CD4^+$ T cells stimulated by P-Mo was determined by $^3$H-thymidine incorporation (0.5 μCi/well; Sp. Ac. 5 Ci/mmol; Radiochemical Centre, Amersham Co., Oakville, Ontario, Canada). Assays were performed in triplicate and the radioactivity was expressed as the mean count per minute (cpm) with standard deviation (S.D.). The results are given as a stimulation index (SI), calculated as in Equation 2.

$$SI = \frac{\text{cpm of CD4}^{+}\text{ T cells + P-Mo(HBsAg)}}{\text{cpm of CD4}^{+}\text{ T cells + Mo(w/oHBsAg)}} \quad (2)$$

*Flowy tometry:* PBMC were fluorescence labelled using, simultaneously, fluorescein (FITC)-conjugated anti-ICAM-1 (CD54, Dianova), or anti-HLA-DR/1A mAb and phycoerythrin (PE)-labelled anti-CD14 mAb (Becton–Dickinson). Analysis was performed by flow cytometry (FACScan, Becton–Dickinson) in double colour, direct fluorescence. The instrument was calibrated with inert beads to which a known number of fluorescein molecules were covalently bound (Flow Cytometry Standards Corp., Research Triangle Park, NC).17 Data were analysed using Consort-30 software (Becton–Dickinson). Receptor expression was presented as receptor density (RD) on the cell surface, shown as the mean fluorescence intensity (MFI) in arbitrary units. MFI was proportional to the number of receptor binding sites on the cell surface.

*Statistical analysis:* All data were analysed by using Student's paired *t*-test (Statgraphics software package). Non-parametric equivalents of this test (Kruskal–Wallis and Wilcoxon matched-pair rank sum test) were also performed to confirm the results. The significance level was $p < 0.05$.

### Results

*Internalization activity of monocytes:* Internalization of the HBsAg preparation by monocytes was measured by assessing phagocytic activity, using flow cytometry (Fig. 1). In ESRD patients the mean number of particles containing HBsAg phagocytes by monocytes was significantly decreased compared with that in controls ($p < 0.001$). Moreover, the mean value of internalized molecules containing HBsAg was especially low in uraemic non-responders compared with the value in uraemic responders ($p < 0.05$) and in controls ($p < 0.001$). There were no differences in phagocytic (internalization) activity between ad- and ay-subtypes of HBsAg. However, the pure HBsAg preparation was much less internalized in all groups of examined patients and controls than was the HBsAg complexed with IgG; the best internalization was observed by using specific immune complexes (IC–HBsAg–IgG) isolated from patients suffering from HBV related glomerulonephritis. The monocytes are additionally able to bind IC-HBsAg–IgG by their receptors to the Fc fragment of IgG, as well as to the $C_{1q}$ receptors. Hence, this facilitation of internalization processes has to be taken into account.

*Expression of adhesion and accessory molecules (ICAM-1; HLA–DR/Ia) on monocytes:* The percentage of positive cells with regard to the defined receptors (ICAM-1; HLA–DR/Ia) revealed no significant differences compared with controls. The expression of accessory and adhesion molecules was interpreted as RD. On freshly isolated uraemic monocytes the density of ICAM-1 (CD54) and HLA-DR/Ia molecules was not significantly decreased ($p > 0.05$) (Table 1).

Induction of monocytes for 18 h with HBsAg as a soluble protein (Table 1) resulted in an increased HLA–DR/Ia expression both on uraemic and control monocytes. Simultaneously, the differences between non-responders and responders in the ESRD patient group were enhanced. ICAM-1 density was also higher compared with that on freshly isolated monocytes, but no differences between uraemic patients and controls could be detected.

When monocytes were cultured with $CD4^{+}$ T lymphocytes these cell-to-cell interactions resulted in a further increase of HLA–DR/Ia and ICAM-1 expression on monocytes but their expression was lower in uraemic non-responders (Table 1).

*Proliferative response of helper-inducer ($CD4^{+}$) T cells induced by autologous monocytes presenting HBsAg:* The proliferative response of $CD4^{+}$ T cells induced by monocytes presenting HBsAg was significantly decreased in ESRD patients ($p < 0.05$), especially in non-responders ($p < 0.001$) (Fig. 2).

### Discussion

Impaired response to HBV vaccination in ESRD patients still remains a pivotal problem in the prevention of HBV infection among the patients and hospital staff in dialysis units.[18–20] Therefore, the present study was focused on some events which are associated with antigen processing by monocytes as APC. We found that in uraemic non-responders to HBV vaccination the internalization of HBsAg by monocytes was significantly decreased; HBsAg was better internalized as the immune complex HBsAg-IgG. In the presentation processes the expression of adhesion molecules (ICAM-1) and accessory molecules (HLA-DR/Ia) was significantly decreased, especially in uraemic non-responders. Impaired internalization of HBsAg, as well as a decrease in ICAM-1 and HLA-DR/Ia expression, correlated well with the blunted proliferation of $CD4^{+}$ T cells stimulated by autologous monocytes induced by HBsAg.

The lack of response to HBV vaccination in ESRD patients may not only be related to uraemic toxicity or intermittent exposure to the haemodialysis environment[2,21] but also seems to depend on individual predisposition, associated with HLA-configura-

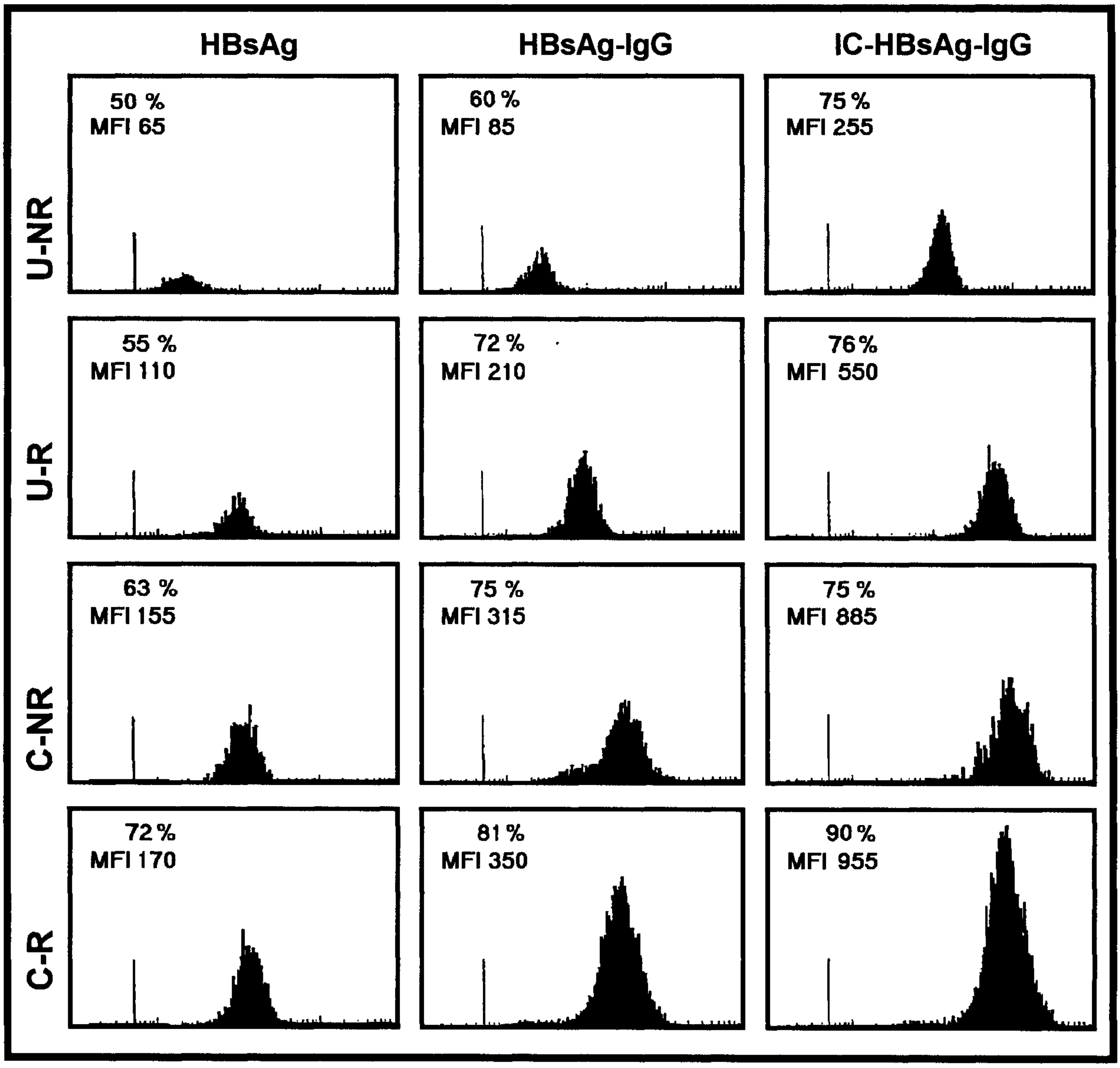

FIG. 1. Internalization capacity of monocytes according to different HBsAg preparations in ESRD patients and controls, in groups of responders and non-responders to hepatitis B vaccination. Experiments were performed with free HBsAg, HBsAg prepared *in vitro* as an immune complex with specific IgG (HBsAg–IgG) and with immune complexes (IC) isolated from serum of patients suffering from glomerulonephritis associated with HBV infection (IC–HBsAg–IgG). Internalization of HBsAg preparations conjugated with FITC was assessed by flow cytometry (FACScan, Becton–Dickinson). The live gate (Consort-30 Software) was estimated on the monocyte cluster by simultaneously defined forward scatter (FSC) and side scatter (SSC) parameters. Internal mean green fluorescence intensity (FL1-axis X) represents the mean number of internalized HBsAg–FITC molecules per cell. Relative cell number (%, axis-Y) indicates the number of monocytes internalizing HBsAg–FITC. U, ESRD patient; C, control; NR, non-responder; R, responder.

tion.[5,6,22,23] Available data concerning the impaired function of monocytes in ESRD patients are based mainly on the fact that addition of monocytes isolated from healthy donors or, alternatively IL-2, restores the inhibited proliferation of T cells in uraemic non-responders.[24–27] Unfortunately, the exact mechanism involved in the process of antigen presentation and recognition has not yet been resolved. Studies performed *in vitro* have revealed a defect of IL-2 secreted by activated T cells,[28,29] while a higher IL-2 receptor expression has been observed in patients not responding to HBV vaccination.[21,30,31]

The dysregulation at the level of the TCR/CD3 receptor complex in T cell–monocytes cooperation might result in an inadequate expression of adhesion molecules (e.g. ICAM-1) as well as accessory or co-stimulatory molecules (e.g. HLA-DR/Ia; CD4).[17] A co-stimulatory signal is essential for the induction of IL-2 synthesis and T cell proliferation. This co-stimulatory activity delivered by monocyte–T cell interactions involves the expression of adhesion molecule ligands (e.g. ICAM-1/LFA-1) as well as the synthesis and release if IL-1 and IL-6.[32,33] Moreover, the preactivation state in uraemia characterized by a high IL-2 receptor (IL-2R) expression and a high density of LFA-1β and CD4 molecules on helper-inducer T cells may be a consequence of impaired monocyte co-stimulatory function.[17,21,30,32–35] In our experimental system, in which $CD4^+$ T cells stimulated by autologous activated monocytes served as APC, the decrease in $CD4^+$ T lymphocyte proliferation in uraemic non-responders not only resulted

**Table 1.** ICAM-1 adhesion molecule expression and expression of HLA-DR/Ia accessory molecule expression on monocytes[a]

| | Before culture | | After 18 h induction with HBsAg | | After 6 days culture with autologous CD4 T cells | |
|---|---|---|---|---|---|---|
| | NR | R | NR | R | NR | R |
| ICAM-1 | | | ② | | ① | |
| | | ③ | | ② | | ② |
| ESRD patients | 180 ± 35[b] | 192 ± 43 | 600 ± 110 | 640 ± 125 | 305 ± 72 | 450 ± 68 |
| | | | | | ② | ① |
| Controls | 250 ± 53 | 305 ± 49 | 490 ± 95 | 510 ± 78 | 682 ± 145 | 835 ± 121 |
| | | ① | | ① | | |
| | | | ① | | ② | |
| HLA-DR/Ia | | | | | | |
| | | | ① | | ① | |
| ESRD patients | 410 ± 120 | 420 ± 110 | 550 ± 121 | 693 ± 193 | 301 ± 135 | 410 ± 92 |
| | | | ② | ① | ③ | ③ |
| Controls | 432 ± 93 | 441 ± 105 | 833 ± 190 | 945 ± 114 | 995 ± 232 | 1115 ± 167 |

[a]Two-colour flow cytometry (Becton–Dickinson, FACScan) was performed by using monoclonal antibodies anti-ICAM-1 and anti-HLA-DR conjugated with FITC and anti-CD14 labelled with PE. Analysis was undertaken before and after the 6 day culture of helper-inducer T cells with autologous monocytes presenting HBsAg. ICAM-1 and HLA-DR/Ia were also estimated on monocytes following induction with HBsAg and directly before institution to the culture.
[b]Molecule expression is presented as their density (RD) on the cell surface and is given in arbitrary units of mean fluorescence intensity (MFI). The MFI of background staining (Simultest control; IgG1–FITC and IgG2–PE) ranged from 10 to 13 arbitrary units. Mean values ± S.D. of 30 experiments are presented. Differences between responders (R) and non-responders (NR) within the groups of ESRD patients or controls.
① $p < 0.05$; ② $p < 0.01$; ③ $p < 0.001$.

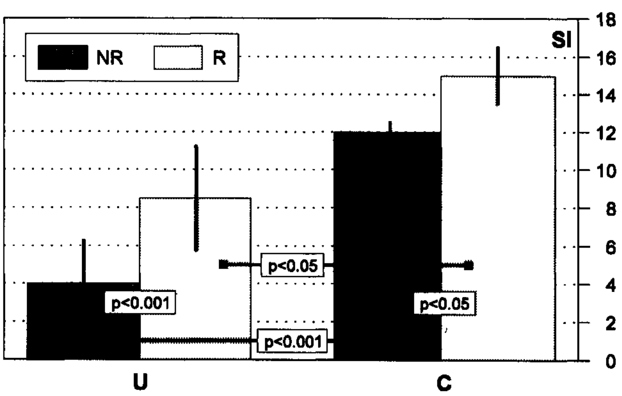

FIG. 2. Proliferative response of helper–inducer (CD4) T cells induced by monocytes presenting HBsAg. Data are expressed as stimulation index (SI). U, uraemic patients; C, controls; NR, non-responders; R, responders. Mean values ± S.D. of 30 experiments are presented.

from impaired IL-2 production, but also from dysregulation in the synthesis of IL-1, IL-6, IL-4 and IFN-γ (data not shown).[5] These findings, associated with a decreased number of IL-1 receptors and IL-6 receptors and a simultaneous increase in IL-2R expression on freshly isolated CD4+ T cells, indicate also that co-stimulation of IL-2-dependent T-cell activation concerning IL-1 and IL-6 activity is impaired.[5,16,32] It is also possible that other factors produced by activated monocytes (e.g. transforming growth factor-β, leukotrienes, prostaglandins-E, TNF-α) may suppress antigen-induced T lymphocyte proliferation.[31,32,36]

Using flow cytometry methods, quantitative determination of monocyte phagocytosis was possible. Monocyte internalization of HBsAg–FITC, measured as phagocytic activity, was significantly decreased in ESRD patients, especially in non-responders. This possibly leads to impaired transformation of this antigen and inadequate presentation to T lymphocytes. However, the use of HBsAg as an immune complex with IgG, and especially as an isolated IC–HBsAg–IgG, improved the blunted monocyte function of antigen internalization, compared with the free HBsAg. This tendency was observed in all groups of patients examined. These immune complexes also resulted in an improvement of ICAM-1 and HLA-DR/Ia expression (data not shown).

The mechanism of impaired phagocytosis and/or internalization observed in uraemia is not well known. However, some authors suggest disorders in ATP content and resting levels of cytosolic calcium ($Ca_i$) in leukocytes[37–39] as well as the negative influence of parathormone on these events. The present study was performed only with leukocytes without separation of monocytes, as APC with regards to HBsAg. Hence, similar disorders in the internalization processes of uraemic monocytes affecting the antigen signalling pathway should be considered.

Adequate antigen presentation and co-stimulatory events are regulated by the TCR/CD3 receptor complex and various cytokines (e.g.TNF-α, IFN-γ).[4,16,33,40,41] For instance, IFN-γ induces increased expression of ICAM-1, which correlates well with enhanced antigen presentation by monocytes. Based on our results in uraemic non-responders the impaired signalling could depend on low ICAM-1 and HLA-DR expression. The decreased production of IFN-γ in ESRD patients, in particular in non-responders (data not shown),[5] may result in blunted antigen presentation of monocytes. It would also be important to know whether the concentration of soluble forms of ICAM-1 in serum and in culture supernatants is changed in the examined group of uraemic patients and controls. Soluble ICAM-1 might regulate the expression of its ligand (LFA-1) on $CD4^+$ T cells as well as serving as a prognostic and diagnostic marker of the inflammatory status of these patients.[42,43]

In conclusion, the parameters examined only partially involve the phenomena concerning monocyte antigen processing and presentation. Additional events should be taken into account for further investigations, e.g. intracellular processing of HBsAg following its internalization into monocytes, and a wider variety of adhesion and accessory molecules. Thus, further analysis of the precise mechanisms could lead to simultaneous administration of different cytokines to improve the impaired response to HBV vaccination in dialysis patients, by providing the co-stimulatory signal.

### References

1. Dumann H, Meuer SC, Koehler H. Uremic serum inhibits monocyte dependent, but not interleukin-2-dependent steps of T cell proliferation. *Nephron* 1990: **56**: 162–165.
2. Dumann H, Meuer SC, Renschin G, Koehler H. Influence of thymopentin on antibody response, and monocyte and T cell function in hemodialysis patients who fail to respond to hepatitis B vaccination. *Nephron* 1990; **55**: 136–140.
3. Gibbons RAS, Martinez OM, Gorovoy MR. Altered monocyte function in uremia. *Immunol Immunopathol* 1990; **56**: 66–80.
4. Langhoff E, Ladefoged J. Accessory cell function in mononuclear cell cultures from uremic patients. *Kidney Int* 1990; **37**: 126–130.
5. Stachowski J, Pollok M, Barth C, Maciejewski J, Baldamus CA. Non-responsiveness to hepatitis B vaccination in hemodialysis patients: association with impaired TCR/CD3 antigen receptor expression regulating costimulatory processes in antigen presentation and recognition. *Nephrol Dial Transpl* 1994; **9**: 144–152.
6. Kraemer A, Herth D, Keyserling HJ, *et al.* Non-responsiveness to hepatitis B vaccination: revaccination and immunogenetic typing. *Klin Wochenschr* 1988; **66**: 670–674.
7. Aloisi RM. *Principles of Immunodiagnostics.* St Louis: C.V. Mosby Company, 1979: Chap 8.
8. Goldman M. *Fluorescent Antibody Methods.* New York, Academic Press 1968.
9. Hardy RR. Purification and coupling of fluorescent proteins for use in flow cytometry: In: Weir DM, Herzenberg LA, Blackwell C, Herzenberg LA, eds. *Handbook of Experimental Immunology Vol 1. Immunochemistry.* Oxford: Blackwell Scientific Publications, 1986.
10. Lerner RA, Green N, Alexander H, Fu-Tong Liu, Sutcliffe JG, Sinnick TM. Chemically synthesized peptides predicted from the nucleotide sequence of the hepatitis B virus genome elicit antibodies reactive with the native envelope protein of Dane particles. *Proc Natl Acad Sci* 1982; **78**: 3403–3407.
11. Pasek M, Goto T, Gilbert W, *et al.* Hepatitis B virus genes and their expression in *E. coli. Nature* 1979; **282**: 575–579.
12. Brown SE, Howard CR, Zuckerman AJ, Steward MW. Determination of the affinity of antibodies to hepatitis B surface antigen in human sera. *J Immunol Meth* 1984; **72**: 41–48.
13. Stachowski J, Michałkiewicz J, Gregorek H, Madaliński K, Maciejewski J. Influence of immune complexes containing HBsAg and HBeAg on IL-2 dependent human lymphocyte proliferation. *Allerg Immunol* 1990; **36**: 47–60.
14. Gregorek H, Jung H, Ulanowicz G, Madaliński K. Immune complexes of HBeAg and HBsAg in sera of children with HBV-mediated glomerulonephrotis. *Arch Immunol Ther* 1986; **5**: 73–83.
15. Bouffard P, Lamelin J-P, Zoulim F, Pichoud C, Trepo C. Different forms of hepatitis B virus DNA and expression of HBV antigens in peripheral blood mononuclear cells in chronic hepatitis B. *J Med Virol* 1990; **31**: 312–317.
16. Geppert TD, Davis LS, Gur H, Wacholtz MC, Lipski PE. Accessory cell signals involved in T cell activation. *Immunol Rev* 1990; **117**: 5–66.
17. Stachowski J, Pollok M, Burrichter H, Spithaler C, Baldamus CA. Signalling via TCR/CD3 antigen receptor complex in uremia is limited by receptors number. *Nephron* 1993; **64**: 369–375.
18. Johnson RJ, Couser WG. Hepatitis B infection and renal disease: clinical, immunopathogenetic and therapeutic considerations. *Kidney Int* 1990; **37**: 663–676.
19. Juszczyk J. Immunopathology of hepatitis B virus infection. *Przeg Epid* 1989; **43**: 177–191.
20. Koehler H, Dumann H, Meyer zum Bueschenfelde K-H, Meuer S. Sekundaerer Immunodefekt bei Niereninsuffizienz am Beispiel der Hepatitis B-Impfung. *Klin Wochenschr* 1988; **66**: 865–872.
21. Dumann H, Meuer SC, Meyer zum Bueschenfelde K-H, Koehler H. Hepatitis B vaccination and interleukin-2 receptor expression in chronic renal failure. *Kidney Int* 1990; **38**: 1164–1168.
22. Alper CA, Kruskall MS, Marcus-Bagley D, *et al.* Genetic predictions of nonresponse to hepatitis B vaccine. *N Engl J Med* 1989; **14**: 708–712.
23. Pol S, Legendre C, Mattlinger B, Berthelot P, Kreis H. Genetic basis of nonresponse to hepatitis B vaccines in hemodialysis patients. *J Hepatol* 1990; **11**: 385–387.
24. Meuer SC, Dumann H, Meyer zum Bischenfelde K-H, Koehler H. Low-dose interleukin-2 induces systemic immune responses against HBsAg in immunodeficient non-responders to hepatitis B vaccination. *Lancet* 1989; **7**: 15–17.
25. Meuer SC, Hauer M, Kurz P, Meyer zum Bueschenfelde K-H, Koehler H. Selective blockade of the antigen-receptor mediated pathway of T cell activation in patients with impaired primary immune responses. *J Clin Invest* 1987; **80**: 743–749.
26. Meuer SC, Koehler H, Meyer zum Bueschenfelde K-H. Monocyte defect in hemodialysis patients supports nonresponsiveness to hepatitis B vaccination and development of HBsAg carrier state. In: Zuckermann AI, ed. *Viral Hepatitis and Liver Disease.* New York: Liss, 1988, 711–713.
27. Zeitz M. Hepatitis-B-Impfung bei Dialysepatienten: Reversibilitaet der defekten Immunantwort durch gleichzeitige Gabe von Interleukin-2. *Z Gastroenterol* 1990; **28**: 179–180.
28. Langhoff E, Hofmann B, Odum N, Ladefoged J, Platz P, Svejgaard A. Kinetic analysis of interleukin-2 (IL-2) production and expression of IL-2 receptors by uraemic and normal lymphocytes. *Scand J Immunol* 1987; **25**: 29–36.
29. Raskova J, Ghobrial I, Ghea SM, Ebert EC, Eisinger R, Raska K. T cells in patients undergoing chronic hemodialisis: mitogenic response, suppressor activity, and interleukin-2 production and receptor generation. *Diagn Immunol* 1986; **4**: 208–216.
30. Beaurain G, Naret C, Marcon L, *et al. In vitro* T cell preactivation in chronic uremic hemodialysed and non-dialysed patients. *Kidney Int* 1989; **36**: 636–644.
31. Desmyter J, Ceuppens JL. Effect of prostaglandin synthesis inhibition *in vivo* on the immune response following hepatitis B vaccination in normal subjects. *Int J Immunopharmacol* 1987; **9**: 803–810.
32. Altman A, Coggeshall KM, Mustelin T. Molecular events mediating T cell activation. *Adv Immunol* 1989; **48**: 227–353.
33. Springer TA. Adhesion receptors of the immune system. *Nature* 1990; **346**: 425–434.
34. Stachowski J, Pollok M, Burrichter H, Baldamus CA. Immunodeficiency in ESRD-patients is linked to altered IL-2 receptor density on T cell subsets. *J Clin Lab Immunol* 1991; **34**: 171–177.
35. Tsakolos ND, Theoharides TC, Hendler ED, Goffinet J, Dwyer JM, Whisler RL, Askenase PW. Immune defects in chronic renal impairment: evidence for defective regulation of lymphocyte response by macrophages from patients with chronic renal impairment on haemodialysis. *Clin Exp Immunol* 1986; **63**: 218–227.
36. Assoian RK, Fleurdelys BE, Stevenson HC, *et al.* Expression and secretion of type β transforming growth factor by activated human macrophages. *Proc Natl Acad Sci USA* 1987; **84**: 6020–6024.
37. Alexiewicz JM, Smogorzewski M, Fadda GZ, Massry SG. Impaired phagocytosis in dialysis patients: studies on mechanisms. *Am J Nephrol* 1991; **11**: 102–111.
38. Franco A, Barnaba V, Ruberti G, Benvenuto R, Balsano C, Musca A. Liver-derived T cell clones in autoimmune chronic active hepatitis: accessory cell function of hepatocytes expressing class II major histocompatibility complex molecules. *Clin Immunol Immunopathol* 1990; **54**: 382–394.
39. Ruiz P, Gomez F, Schreiber AD. Impaired function of macrophage Fc receptors in end-stage renal disease. *N Engl J Med* 1990; **322**: 711–722.
40. Quiroga JA, Castillo I, Porres JC, *et al.* Recombinant γ-interferon as adjuvant to hepatitis B vaccine in hemodialysis patients. *Hepatology* 1990; **12**: 661–663.
41. Stachowski J, Pollok M, Burrichter H, Spithaler C, Baldamus CA. Does uremic environment down-regulate T cell activation via TCR/CD3 antigen receptor complex? *J Clin Lab Immunol* 1991; **36**: 15–21.
42. Becker JC, Dummer R, Hartman AA, Burg G, Schmidt RE. Shedding of ICAM-1 from human melanoma cell lines induced by IFN-gamma and tumor necrosis factor alpha. Functional consequences on cell-mediated cytotoxicity. *J Immunol* 1991; **147**: 4398–4401.
43. Seth R, Raymond FD, Makgoda MW. Circulating ICAM-1 isoforms: diagnostic prospects for inflammatory and immune disorders. *Lancet* 1991; **338**: 83–84.

ACKNOWLEDGEMENTS. This work was supported by a research grant from Else Kroener Fresenius Foundation (Germany), and in part by grant Nos PB 0210-S/4/-93-05 and PB 4-S 405 005 04 from the Committee for Scientific Research (Poland). These investigations were, in part, presented at the 27th Annual Meeting of European Society for Paediatric Nephrology, 5–8 September 1993, Heidelberg, Germany and at the 31st Annual Congress of the European Dialysis and Transplant Association–European Renal Association (EDTA), 3–6 July 1994, Vienna, Austria (*Nephrol Dial Transpl* 1994; **9**: 964). The authors wish to acknowledge Mr Janusz Rakalski (CADr s.c.) for help in preparing the manuscript. We are grateful to Ms Elanne Smootz for her language assistance and to Ms Maria Lewandowska-Stachowiak for her technical assistance.

**Received 1 September 1994; accepted in revised form 14 October 1994**
